# Tempol, a Superoxide Dismutase Mimetic Agent, Inhibits Superoxide Anion-Induced Inflammatory Pain in Mice

**DOI:** 10.1155/2017/9584819

**Published:** 2017-05-14

**Authors:** Catia C. F. Bernardy, Ana C. Zarpelon, Felipe A. Pinho-Ribeiro, Cássia Calixto-Campos, Thacyana T. Carvalho, Victor Fattori, Sergio M. Borghi, Rubia Casagrande, Waldiceu A. Verri

**Affiliations:** ^1^Department of Nursing, Health Science Centre, State University of Londrina, Londrina, PR, Brazil; ^2^Department of Pathology, Biological Science Centre, State University of Londrina, Londrina, PR, Brazil; ^3^Department of Pharmaceutical Sciences, Health Science Centre, State University of Londrina, Londrina, PR, Brazil

## Abstract

The present study evaluated the anti-inflammatory and analgesic effects of the superoxide dismutase mimetic agent tempol in superoxide anion-induced pain and inflammation. Mice were treated intraperitoneally with tempol (10–100 mg/kg) 40 min before the intraplantar injection of a superoxide anion donor, potassium superoxide (KO_2_, 30 *μ*g). Mechanical hyperalgesia and thermal hyperalgesia, paw edema, and mRNA expression of peripheral and spinal cord mediators involved in inflammatory pain, TNF*α*, IL-1*β*, IL-10, COX-2, preproET-1, gp91^phox^, Nrf2, GFAP, and Iba-1, were evaluated. Peripheral and spinal cord reductions of antioxidant defenses and superoxide anion were also assessed. Tempol reduced KO_2_-induced mechanical hyperalgesia and thermal hyperalgesia and paw edema. The increased mRNA expression of the evaluated mediators and oxidative stress in the paw skin and spinal cord and increased mRNA expression of glial markers in the spinal cord induced by KO_2_ were successfully inhibited by tempol. KO_2_-induced reduction in Nrf2 mRNA expression in paw skin and spinal cord was also reverted by tempol. Corroborating the effect of tempol in the KO_2_ model, tempol also inhibited carrageenan and CFA inflammatory hyperalgesia. The present study demonstrates that tempol inhibits superoxide anion-induced molecular and behavioral alterations, indicating that tempol deserves further preclinical studies as a promising analgesic and anti-inflammatory molecule for the treatment of inflammatory pain.

## 1. Introduction

Superoxide dismutase (SOD) is an antioxidant enzyme that regulates the levels of reactive oxygen species such as superoxide anion (O_2_^•−^) under basal conditions in tissue metabolism and health [1, 2]. However, reactive oxygen species may shatter healthy tissues by itself or through interactions with other molecules such as nitric oxide (NO) in inflammation. During inflammatory reactions, free radicals are produced at rates which overwhelm the endogenous antioxidant capacity, making the tissues vulnerable to injuries.

Superoxide anion is the initial product of oxygen reduction and its inhibition represents an important step to prevent the deleterious events related to oxidative stress [3, 4]. After tissue injury and/or infections, neutrophils are initially recruited following inflammatory and chemotactic signals, which include the inflammatory cytokines interleukin-1 beta (IL-1*β*) and tumor necrosis factor (TNF) [5] as well as reactive oxygen species themselves [6]. After being recruited into the inflammatory focus, neutrophils are activated by these signals, including the activation of the so-called respiratory burst represented by the nicotinamide adenine dinucleotide phosphate (NADPH) oxidase membrane-bound enzyme complex production of superoxide anion in neutrophils. This scenario characterized by the exacerbation of superoxide anion production propagates the inflammatory response resulting in sensitization of primary nociceptive neurons [7]. The terminal endings of nociceptive neurons are widespread in all tissues, where they identify noxious (intense) stimuli, including trauma, chemicals, heat, and cold. In fact, reactive oxygen species and cytokines sensitize and activate nociceptive neurons during the inflammatory response [8–10]. Interestingly, the nociceptive transmission is enhanced by reactive oxygen and nitrogen species not only at the site of initial injury, but also at the spinal cord level in response to increased nociceptive transmission after a peripheral injury [1]. In this sense, targeting superoxide anion and its downstream effectors at peripheral and central sites is a promising approach to inhibit inflammatory pain.

Tempol (4-hydroxy-2,2,6,6-tetramethylpiperidine-*N*-oxyl) is a redox-cycling nitroxide water-soluble SOD mimetic agent. Due to its low molecular weight, tempol passes through biological membranes [11]. Tempol favors the metabolism of a wide variety of cellular reactive oxygen and nitrogen species and reduces oxidative stress. Thus, tempol is a potent antioxidant [12]. The beneficial biological effects of tempol range from protective effects against radiation, metabolic syndrome, and shock as well as heart, kidney, and central nervous system safekeeping [12]. Tempol inhibits superoxide anion-induced neuronal firing, thermal hyperalgesia, and edema induced by carrageenan, neuropathic pain induced by chronic constriction injury (CCI) [13–15] and chemotherapy [16] in rats, and TNF*α*-induced mechanical hyperalgesia and neutrophils recruitment in mice [7]. Tempol treatment also eliminates free radicals and inhibits lipid peroxidation in vitro and in vivo [15, 17–19]. However, the literature lacks a proof-of-concept demonstration that tempol inhibits superoxide anion-triggered pain and inflammation.

In the present study, the effects of SOD mimetic agent tempol were evaluated in a model of superoxide anion donor (potassium superoxide; KO_2_)-induced pain and inflammation in mice.

## 2. Methods

### 2.1. Animals

The experiments were performed on male Balb/c mice (20–25 g) housed in standard plastic cages with free access to food and water. All behavioral testing was performed between 9:00 am and 5:00 pm in a temperature- and light-controlled room. Animals' care and handling procedures were in accordance with the International Association for Study of Pain (IASP) guidelines and with the approval of the Ethics Committee of State University of Londrina (process 7706.2013-42). Mice were used only once and were acclimatized to the testing room at least 1 hour before the experiments. At the end of experiments, mice were anesthetized with isoflurane 3% to minimize suffering (Abbott Park, IL, USA) and terminally killed by cervical dislocation followed by decapitation. Animals were monitored periodically at indicated time points during the experiments. No unexpected animal deaths occurred during this study. All efforts were made to minimize the number of animals used and their suffering.

### 2.2. Drugs and Reagents

Drugs and reagents used in the study were obtained from the following sources: Potassium superoxide (KO_2_) 96.5% was purchased from Alfa Aesar (Ward Hill, MA, USA); 4-hydrophosphinyloxy TEMPO, triethylammonium salt (tempol) 99%, and carrageenan were purchased from Santa Cruz Biotechnology (Santa Cruz, CA, USA); nitroblue tetrazolium (NBT) was purchased from Amresco (Solon, OH, USA); complete Freund's adjuvant (CFA), ferric chloride hexahydrate, 2,4,6-tripyridyl-s-triazine (TPTZ), and ABTS [2,20-azino-bis(3-ethylbenzothiazoline-6-sulfonate)] were purchased from Sigma-Aldrich (St. Louis, MO, USA).

### 2.3. Experimental Protocols

Mice were treated intraperitoneally (i.p.) with vehicle (sterile saline, 100 *µ*L, i.p.) or tempol (10–100 mg/kg and 100 mg/kg, 100 *µ*L, i.p.) and after 40 min received intraplantar (i.pl.) injection of vehicle (control group, saline, 25 *µ*L) or KO_2_ (30 *µ*g, 25 *µ*L). Mechanical hyperalgesia and thermal hyperalgesia and paw edema measurements were performed between 1 and 5 h after KO_2_ injection. Cytokines (TNF*α*, pro-IL-1*β*, and IL-10), cyclooxygenase-2 (COX-2), and preproendothelin-1 (preproET-1) mRNA expressions were determined in paw tissue and spinal cord (L4–L6) samples 3 h after KO_2_ or vehicle injection. Following the same protocol, antioxidant capacity was evaluated by the ferric reducing ability of plasma (FRAP), ABTS, and glutathione reduced (GSH) levels assay and oxidative stress was evaluated by superoxide anion production (NBT reduction assay) assay in the paw skin and spinal cord tissues. The gp91^phox^ and nuclear factor erythroid 2-related factor (Nrf2) mRNA expressions were evaluated in the paw skin and spinal cord tissues. Glial fibrillary acidic protein (GFAP) and ionized calcium binding adaptor molecule 1 (Iba-1) mRNA expressions were determined in spinal cord samples. In another experiment, mice were treated intraperitoneally (i.p.) with vehicle (sterile saline, 100 *µ*L, i.p.) or tempol (100 mg/kg and 100 mg/kg, 100 *µ*L, i.p.) and after 40 min received intraplantar (i.pl.) injection of vehicle (control group, saline, 25 *µ*L) or carrageenan (300 *µ*g, 25 *µ*L), respectively. Mechanical hyperalgesia and thermal hyperalgesia measurements were performed between 1 and 5 h after carrageenan injection. In a prolonged inflammation model, vehicle or tempol treatment (100 mg/kg, 100 *µ*L, i.p.) was given daily starting one day after CFA (10 *µ*L) i.pl. injection, and mechanical hyperalgesia and thermal hyperalgesia were evaluated always 30 min after treatment from 1 to 7 days. The dose of carrageenan, CFA, and KO_2_ and time points chosen for the experimental protocol were determined according to previous studies of our laboratory [7, 20–23].

### 2.4. Electronic Pressure Meter Test for Mechanical Hyperalgesia Measurement

The test consisted of evoking a hindpaw reflex with a handheld force transducer (Electronic von Frey Anesthesiometer; Insight, Ribeirão Preto, SP, Brazil) adapted with a 0.5 mm^2^ polypropylene tip. Detailed methodology was previously described [24]. The results are expressed by delta (Δ) of withdrawal threshold (in g) calculated by subtracting the measurements at the indicated time points after stimulus injection from the basal measurements.

### 2.5. Hot Plate Test

The test was performed as reported previously [25]. Briefly, mice were placed the hot plate apparatus (EFF 361, Insight Equipamentos, Ribeirão Preto, SP, Brazil) maintained at 55°C. The reaction time was scored when hindpaw licking or flinching occurred in the injected paw and/or mice jumped. As the basal latencies were always smaller than 20 s (between 15 and 18 s), a maximum latency (cut-off) was set at 20 s to avoid tissue damage.

### 2.6. Paw Edema

The paw edema formation was measured using a caliper (Digimatic Caliper, Mitutoyo Corporation, Kanagawa, Japan). Values of paw thickness are expressed as the difference (Δ mm) between the values obtained just before (basal) and after stimulus injection.

### 2.7. Reverse Transcriptase and Quantitative Polymerase Chain Reaction (RT-qPCR)

Paw skin and spinal cord tissue samples were collected 3 h after KO_2_ or vehicle injection and homogenized in TRIzol® reagent. The total RNA was extracted using the SV Total RNA Isolation System (Promega Biosciences, Fitchburg, WI, USA). RT-PCR and quantitative PCR were performed using GoTaq® 2-Step RT-qPCR System (Promega) following the manufacturer's directions. Complementary DNA was reverse-transcribed from 2 *μ*g of total RNA, and quantitative PCR was performed on a LightCycler® Nano Instrument (Roche). The following primer sequences were used: TNF*α*: sense, 5′-TCTCATCAGTTCTATGGCCC-3′, antisense, 5′-GGGAGTAGACAAGGTACAAC-3′; pro-IL-1*β*: sense, 5′-GAAATGCCACCTTTTGACAGTG-3′, antisense, 5′-TGGATGCTCTCATCAGGACAG-3′; IL-10: sense, 5′-TCTCATCAGTTCTATGGCCC-3′; antisense, 5′-GGGAGTAGACAAGGTACAAC-3′; COX-2: sense, 5′-GTGGAAAAACCTCGTCCAGA-3′, antisense, 5′-GCTCGGCTTCCAGTATTGAG-3′; preproET-1: sense, 5′-TGTGTCTACTTCTGCCACCT-3′, antisense, 5′-CACCAGCTGCTGATAGATAC-3′; gp91^phox^: sense, 5′-AGCTATGAGGTGGTGATGTTAGTGG-3′, antisense, 5′-CACAATATTTGTACCAGACAGACTTGAG-3′; Nrf2: sense, 5′-TCACACGAGATGAGCTTAGGGCAA-3′, antisense, 5′–TACAGTTCTGGGCGGCGACTTTAT-3′; GFAP: sense, 5′-GGCGCTCAATGCTGGCTTCA-3′, antisense, 5′-TCTGCCTCCAGCCTCAGGTT-3′; Iba-1: sense, 5′-ATGGAGTTTGATCTGAATGGAAAT-3′, antisense, 5′-TCAGGGCAGCTCGGAGATAGCTTT-3′; and GAPDH: sense, 5′-CATACCAGGAAATGAGCTTG-3′, antisense, 5′-ATGACATCAAGAAGGTGGTG-3′. The expression of GAPDH mRNA was used as a reference gene to normalize data.

### 2.8. FRAP Assay

Mice were euthanized and samples of paw skin tissue and spinal cord were collected 3 h after KO_2_ or vehicle injection. The ability of paw skin and spinal cord tissues to reduce ferric ion was determined by FRAP assay [26, 27]. The samples (30 mg) were homogenized in 500 *μ*L of KCl (1.15%) using a Tissue-Tearor (BioSpec Products, Bartlesville, OK, USA) and centrifuged (1.000*g*, 10 min, 4°C), and the supernatant was used to measure the antioxidant capacity of samples. The supernatant (30 *μ*L) was mixed with the FRAP reagent (0.3 mM acetate buffer pH 3.6, 10 mM TPTZ in 40 mM hydrochloride acid, and 20 mM ferric chloride) and incubated at 37°C for 30 min. The absorbance was determined in 595 nm (Multiskan GO, Thermo Fischer Scientific, Vantaa, Finland). Previously, a curve of Trolox was prepared and the results are presented as nanomoles of Trolox equivalent per milligram of tissue.

### 2.9. ABTS Assay

Mice were euthanized and tissue samples of paw skin and spinal cord were collected 3 h after KO_2_ or vehicle injection. The ability of samples to resist oxidative damage was determined by their ability to scavenge the ABTS radical. The supernatant was prepared as for the measurement of the antioxidant capacity in the FRAP assay. The ABTS solution was prepared with 7 mM of ABTS and 2.45 mM of potassium persulfate diluted with phosphate buffer pH 7.4 to absorbance of 0.7–0.8 in 730 nm. Then, the supernatant was mixed with ABTS solution and after 6 min the absorbance was determined in 730 nm (Multiskan GO, Thermo Fischer Scientific, Vantaa, Finland) [26, 27]. A curve of Trolox was prepared and the results are presented as nanomoles of Trolox equivalent per milligram of tissue.

### 2.10. GSH Assay

Paw skin and spinal cord samples of mice were collected 3 h after KO_2_ or vehicle injection and homogenized in ethylenediaminetetraacetic acid (EDTA) 0.02 M and homogenates treated with 2 mL H_2_O Milli Q plus 0.5 mL of trichloroacetic acid (TCA) 50%. In the next step, homogenates were centrifuged (1.500*g*, 15 min) and the resultant supernatant was added to 2 mL of a solution containing Tris 0.4 M (pH 8.9) plus 50 mL of dithionitrobenzoic acid (DTNB). Then, after 5 min, the measurements were performed using a spectrophotometer at 412 nm (Multiskan GO Microplate Spectrophotometer, Thermo Fischer Scientific, Vantaa, Finland) [27]. The results were presented as GSH (mmols per milligrams of tissue).

### 2.11. Superoxide Anion Production

Mice were euthanized and tissue samples of paw skin and spinal cord were collected 3 h after KO_2_ or vehicle injection. The measurement of superoxide anion production in tissue homogenates was performed using the NBT assay as described previously [28]. Briefly, 50 *μ*L of homogenates was incubated with 100 *μ*L of NBT (1 mg/ml) in 96-well plate for 15 min. The supernatant was then carefully removed and reduced formazan solubilized by adding 120 *μ*L of KOH 2 M and 120 *μ*L of dimethylsulfoxide (DMSO). Reduction of NBT to formazan was measured at 600 nm using a microplate spectrophotometer reader (Multiskan GO, Thermo Fischer Scientific, Vantaa, Finland). The tissue weight was used for data normalization and the results are expressed as NBT reduction as optical density per mg of tissue.

### 2.12. Statistical Analysis

Results are presented as means ± SEM of measurements made on 6 mice per group per experiment and are representative of 2 separate experiments. Two-way analysis of variance (ANOVA) was used to compare the groups and doses at all times (curves). The analyzed factors were treatments, time, and time versus treatment interaction. When there was a significant time versus treatment interaction, one-way ANOVA followed by Tukey's *t*-test was performed for each time. Statistical differences were considered to be significant at *p* < 0.05.

## 3. Results

### 3.1. Tempol Inhibits KO_2_-Induced Hyperalgesia and Paw Edema

**Figure 1:**
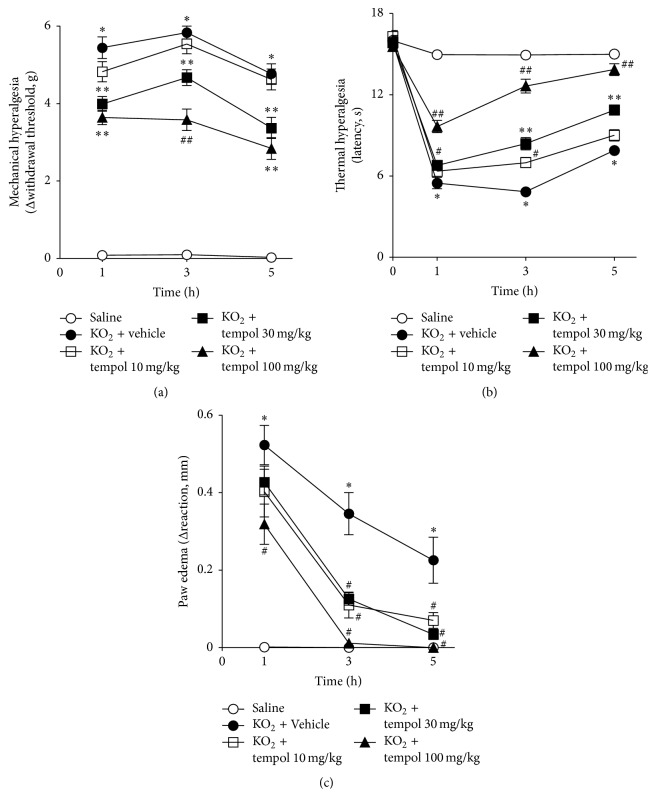
Tempol inhibits KO_2_-induced hyperalgesia and paw edema. Mice were treated with tempol (10–100 mg/kg) or vehicle 40 min before KO_2_ intraplantar injection. Nociceptive thresholds to mechanical (a) and thermal (b) stimuli as well as paw edema (c) were measured 1–5 h after KO_2_ injection. Results are expressed as means ± SEM (*n* = 6 per group per experiment, representative of two separate experiments) [^*∗*^*p* < 0.05 versus saline; ^#^*p* < 0.05 versus KO_2_ + vehicle; ^*∗∗*^*p* < 0.05 versus KO_2_ + tempol 10 mg/kg; ^##^*p* < 0.05 versus KO_2_ + tempol 30 mg/kg (ANOVA followed by Tukey's *t*-test)].

In the first experimental setting, a dose-response curve of potential analgesic and anti-inflammatory effects of tempol was investigated in KO_2_-induced paw inflammation. Mice were treated with 10, 30, and 100 mg/kg of tempol 40 min before the intraplantar injection of KO_2_, and mechanical hyperalgesia, thermal hyperalgesia, and paw edema were evaluated at indicated time points (Figure 1). KO_2_ induced mechanical hyperalgesia (Figure 1(a)) and thermal hyperalgesia (Figure 1(b)) and paw edema (Figure 1(c)) between 1 and 5 h after injection compared to the vehicle group, which were inhibited by tempol in a time- and dose-dependent manner. The dose of 10 mg/kg was inefficient to inhibit KO_2_-induced mechanical hyperalgesia (Figure 1(a)) but partially inhibited thermal hyperalgesia (3 h) (Figure 1(b)) and paw edema (3 and 5 h) (Figure 1(c)). Treatment with the dose of 30 mg/kg fully inhibited KO_2_-induced mechanical hyperalgesia and thermal hyperalgesia (Figures 1(a) and 1(b)) and inhibited KO_2_-induced paw edema at 3 and 5 h (Figure 1(c)). The dose of 100 mg/kg completely inhibited mechanical hyperalgesia and thermal hyperalgesia and paw edema between 1 and 5 h (Figures 1(a)–1(c)) with statistical differences compared with the doses of 10 and 30 mg/kg in mechanical hyperalgesia (3 h) and thermal hyperalgesia (1–5 h) (Figures 1(a) and 1(b)). Thus, the dose of 100 mg/kg of tempol was selected for the next series of experiments.

### 3.2. Tempol Inhibits KO_2_-Induced TNF*α*, IL-1*β*, and IL-10 mRNA Expressions

**Figure 2:**
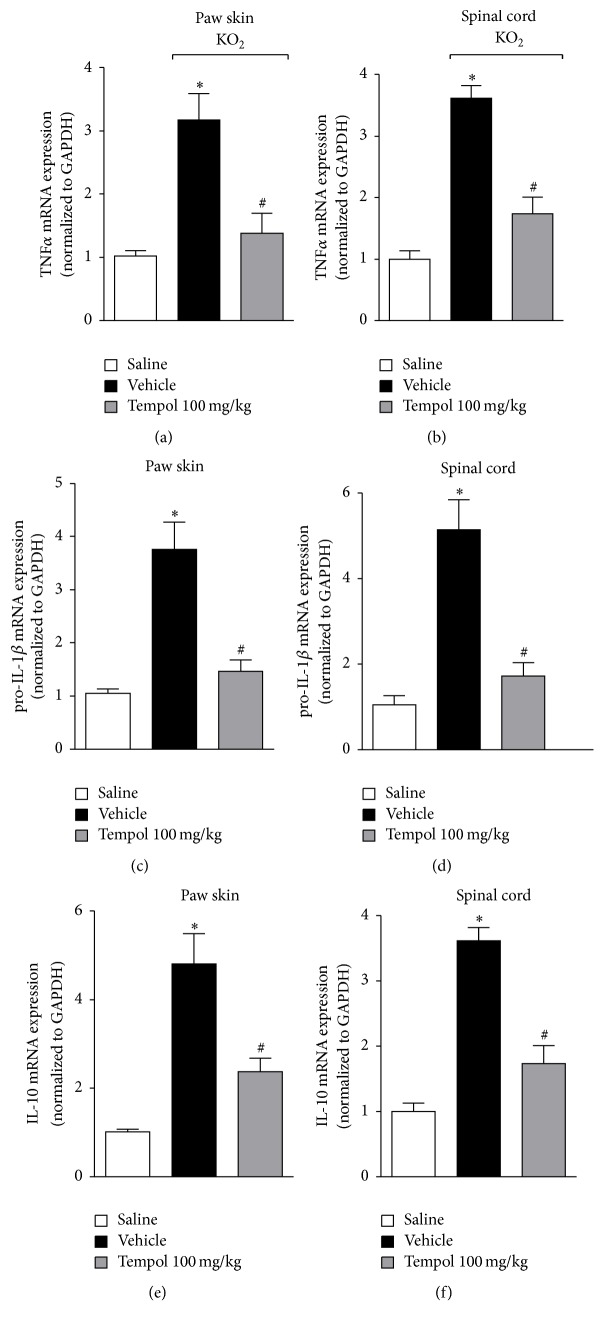
Tempol inhibits KO_2_-induced TNF*α*, IL-1*β*, and IL-10 mRNA expression in the paw skin and spinal cord. Mice were treated with tempol (100 mg/kg) or vehicle 40 min before KO_2_ intraplantar injection. Paw skin and spinal cord samples were collected 3 h after KO_2_-stimulus and TNF*α* ((a) and (b)), IL-1*β* ((c) and (d)), and IL-10 ((e) and (f)) mRNA expressions were evaluated by RT-qPCR. Results were normalized using GAPDH as gene control and expressed as means ± SEM (*n* = 6 per group per experiment, representative of two separate experiments) [^*∗*^*p* < 0.05 versus saline; ^#^*p* < 0.05 versus KO_2_ + vehicle (one-way ANOVA followed by Tukey's *t*-test)].

Cytokines are classical molecules involved in the modulation of inflammatory pain [29]. Prohyperalgesic cytokines such as TNF*α* and IL-1*β* contribute to nociceptor neuron sensitization [7, 30] and cytokines such as IL-10 limit the development of hyperalgesia by inhibiting the production of hyperalgesic cytokines [31]. Therefore, the effect of tempol on KO_2_-induced cytokine production was evaluated. Mice were treated with tempol (100 mg/kg) 40 min before KO_2_ intraplantar injection and after 3 h paw skin and spinal cord samples were collected for TNF*α*, IL-1*β*, and IL-10 mRNA expressions determination by RT-qPCR (Figure 2). KO_2_ induced an increase in TNF*α*, IL-1*β*, and IL-10 mRNA expressions in the paw skin (Figures 2(a), 2(c), and 2(e)) and spinal cord samples (Figures 2(b), 2(d), and 2(f)), which were significantly inhibited by tempol treatment. These data suggest that the analgesic effect of tempol is in part dependent on inhibiting cytokine production.

### 3.3. Tempol Inhibits the KO_2_-Induced COX-2 and preproET-1 mRNA Expressions

**Figure 3:**
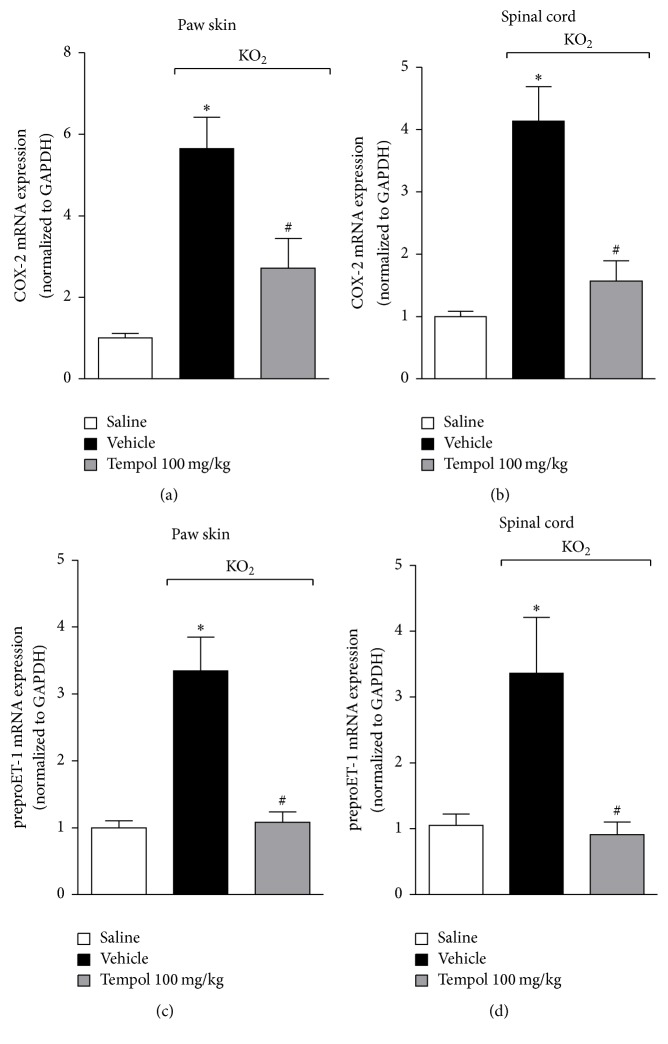
Tempol inhibits KO_2_-induced COX-2 and preproET-1 mRNA expressions in the paw skin and spinal cord. Mice were treated with tempol (100 mg/kg) or vehicle 40 min before KO_2_ intraplantar injection. Paw skin and spinal cord samples were collected 3 h after KO_2_-stimulus and COX-2 ((a) and (b)) and preproET-1 ((c) and (d)) mRNA expressions were evaluated by RT-qPCR. Results were normalized using GAPDH as gene control and expressed as means ± SEM (*n* = 6 per group per experiment, representative of two separate experiments) [^*∗*^*p* < 0.05 versus saline; ^#^*p* < 0.05 versus KO_2_ + vehicle (one-way ANOVA followed by Tukey's *t*-test)].

COX-2 and endothelin-1 are involved in the molecular mechanism of nociceptor neuron sensitization in inflammatory pain. Cytokines induce the production of ET-1, which, in turn, induces the production of COX-2-derived prostaglandin E_2_ (PGE_2_) [29, 32, 33]. PGE_2_ sensitizes nociceptor sensory neurons via protein kinase A (PKA) phosphorylation of sodium channels [30]. Furthermore, KO_2_-induced pain depends on ET-1 [21, 22] and COX-2 [20]. Thus, it is conceivable to evaluate whether tempol inhibits KO_2_-induced mRNA expression of COX-2 and preproET-1. Mice received tempol (as for Figure 2) 40 min before KO_2_ intraplantar injection, and after additional 3 h paw skin and spinal cord samples were collected for COX-2 and preproET-1 mRNA expressions determination by RT-qPCR (Figure 3). Treatment with tempol significantly inhibited KO_2_-induced COX-2 (Figures 3(a) and 3(b)) and preproET-1 (Figures 3(c) and 3(d)) mRNA expressions in the paw skin and spinal cord samples. Thus, inhibiting KO_2_-induced mRNA expression of COX-2 and preproET-1 is a contributing mechanism to the analgesic effect of tempol.

### 3.4. Tempol Improves KO_2_-Induced Depletion of Antioxidant Capacity

**Figure 4:**
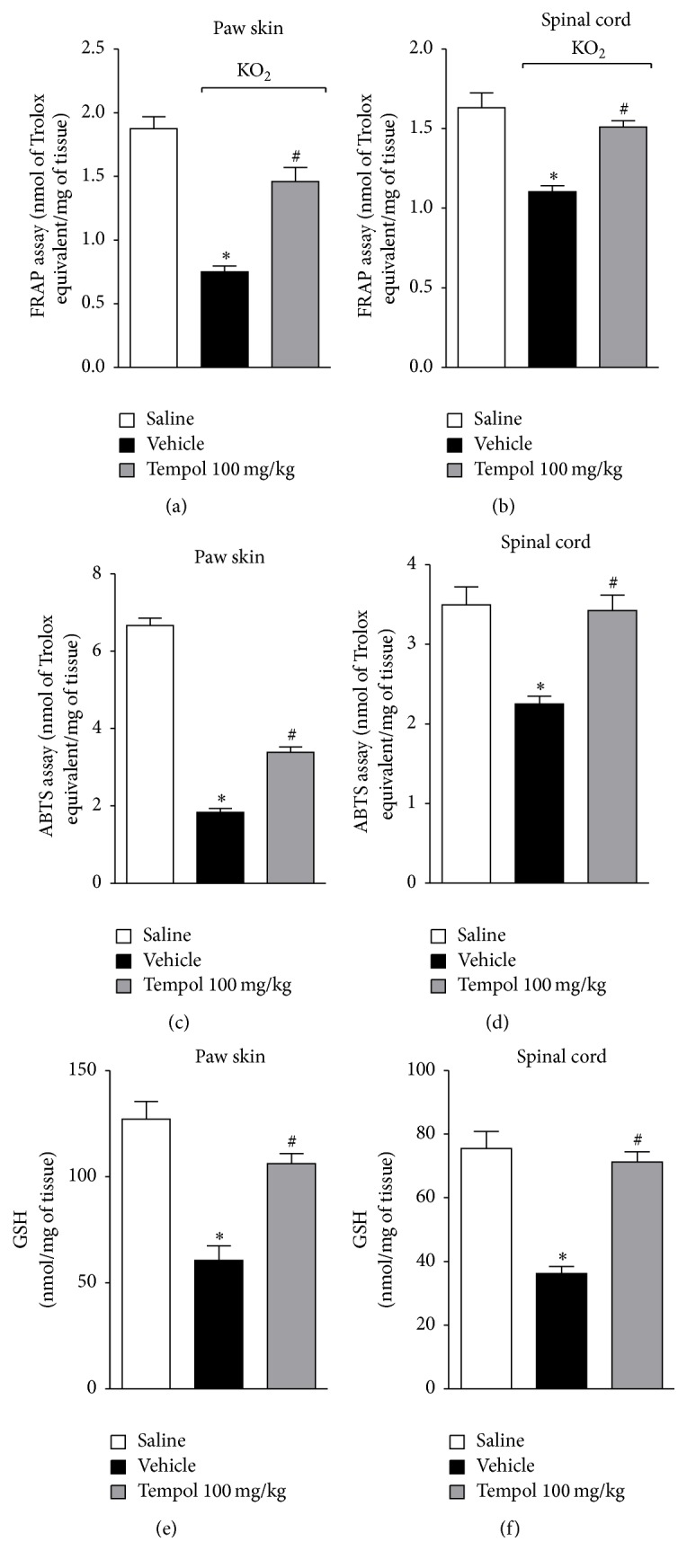
Tempol inhibits KO_2_-induced depletion of antioxidant capacity in the paw skin and spinal cord. Mice were treated with tempol (100 mg/kg) or vehicle 40 min before KO_2_ intraplantar injection. Paw skin and spinal cord samples were collected 3 h after KO_2_-stimulus and the oxidant capacity was evaluated by FRAP ((a) and (b)), ABTS ((c) and (d)), and GSH ((e) and (f)) assays. Results are expressed as means ± SEM (*n* = 6 per group per experiment, representative of two separate experiments) [^*∗*^*p* < 0.05 versus saline; ^#^*p* < 0.05 versus KO_2_ + vehicle (one-way ANOVA followed by Tukey's *t*-test)].

The endogenous antioxidant system is an important mechanism to maintain tissue homeostasis through containment of oxidative stress [2]. In this sense, the implications of tempol upon antioxidant response in KO_2_-induced inflammatory pain should be determined. Mice were treated with tempol (as for Figure 2) 40 min before KO_2_ intraplantar injection, and after additional 3 h paw skin and spinal cord samples were collected for evaluation of antioxidant capacity by FRAP, ABTS, and GSH assays (Figure 4). KO_2_ induced endogenous antioxidant depletion in the paw skin and spinal cord as observed by reduction of FRAP (Figures 4(a) and 4(b)), ABTS (Figures 4(c) and 4(d)), and GSH (Figures 4(e) and 4(f)) assays. On the other hand, treatment with tempol restored the antioxidant capacity of paw skin and spinal cord samples (Figures 4(a)–4(f)). These data provide evidence that preserving the endogenous production of antioxidants is a contributing mechanism of tempol to inhibit KO_2_-induced inflammatory pain.

### 3.5. Tempol Inhibits KO_2_-Induced Oxidative Stress

**Figure 5:**
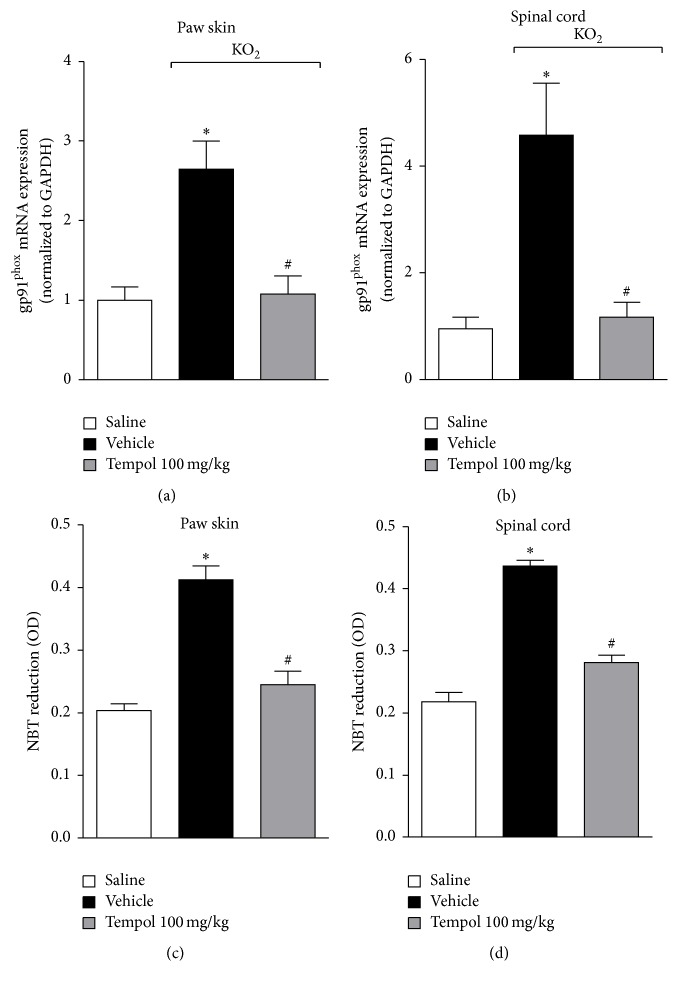
Tempol inhibits KO_2_-induced oxidative stress in the paw skin and spinal cord. Mice were treated with tempol (100 mg/kg) or vehicle 40 min before KO_2_ intraplantar injection. Paw skin and spinal cord samples were collected 3 h after KO_2_ injection to evaluate gp91^phox^ mRNA expression ((a) and (b)) and NBT reduction (superoxide anion production assay, (c) and (d)) by qPCR and colorimetric assay, respectively. Results were normalized using GAPDH as gene control and expressed as means ± SEM (*n* = 6 per group per experiment, representative of two separate experiments) [^*∗*^*p* < 0.05 versus saline; ^#^*p* < 0.05 versus KO_2_ + vehicle (one-way ANOVA followed by Tukey's *t*-test)].

Oxidative stress mediates inflammatory pain [2]. Therefore, to confirm the antioxidant effect of tempol in KO_2_-induced inflammatory pain, the next experiments assessed the mRNA expression of gp91^phox^, a subunit of NADPH oxidase which catalyzes superoxide anion production during inflammation [28], and superoxide anion levels. Mice received tempol (as for Figure 2) 40 min before KO_2_ intraplantar injection, and after additional 3 h paw skin and spinal cord samples were collected for RT-qPCR determination of gp91^phox^ mRNA expression and superoxide anion production (Figure 5). KO_2_-induced gp91^phox^ mRNA expression and superoxide anion production were inhibited by tempol treatment in the paw skin (Figures 5(a) and 5(b)) and spinal cord (Figures 5(c) and 5(d)). The inhibition of KO_2_-induced gp91^phox^ mRNA expression and its product, superoxide anion, demonstrate that tempol inhibits the production and system involved in the production of superoxide anion, therefore contributing to reducing oxidative stress.

### 3.6. Tempol Inhibits KO_2_-Induced Decrease of Nrf2 mRNA Expression

**Figure 6:**
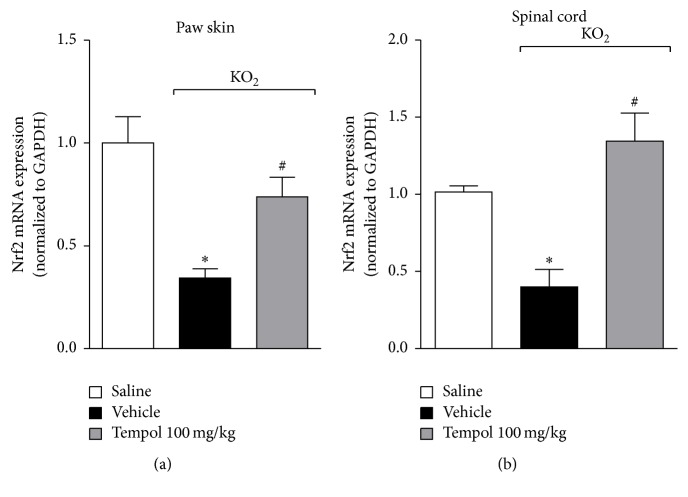
Tempol inhibits KO_2_-induced decrease of Nrf2 mRNA expression in the paw skin and spinal cord. Mice were treated with tempol (100 mg/kg) or vehicle 40 min before KO_2_ intraplantar injection. Paw skin and spinal cord samples were collected 3 h after KO_2_ injection and Nrf2 mRNA expression was evaluated by qPCR. Results were normalized using GAPDH as gene control and expressed as means ± SEM (*n* = 6 per group per experiment, representative of two separate experiments) [^*∗*^*p* < 0.05 versus saline; ^#^*p* < 0.05 versus KO_2_ + vehicle (one-way ANOVA followed by Tukey's *t*-test)].

Other important pathways that control the deleterious impact of oxidative stress in tissues are mediated by the transcription factor Nrf2. KO_2_ reduces Nrf2 mRNA expression and drugs that enhance Nrf2 mRNA expression inhibit KO_2_-induced pain [34]. Thus, the effect of tempol in KO_2_-induced reduction of Nrf2 mRNA expression was investigated. Mice were treated with tempol (as for Figure 2) 40 min before KO_2_ intraplantar injection, and after additional 3 h paw skin and spinal cord samples were collected for the determination of Nrf2 mRNA expression (Figure 6). KO_2_ significantly reduced Nrf2 mRNA expression in the paw skin and spinal cord compared to vehicle group. Tempol treatment prevented the KO_2_-induced reduction of Nrf2 mRNA expression levels (Figures 6(a) and 6(b)). This result suggests that tempol preserves the expression of an endogenous transcription factor involved in controlling KO_2_-induced oxidative stress and inflammatory pain.

### 3.7. Tempol Inhibits KO_2_-Induced Glial Cells Activation

**Figure 7:**
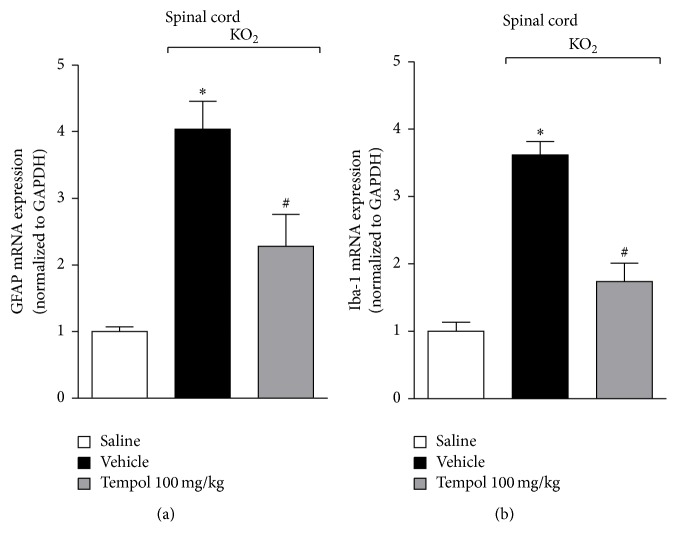
Tempol inhibits KO_2_-induced astrocytes and microglia activation in the spinal cord. Mice were treated with tempol (100 mg/kg) or vehicle 40 min before KO_2_ intraplantar injection. Spinal cord samples were collected 3 h after KO_2_ injection and GFAP (a) and Iba-1 (b) mRNA expressions were evaluated by RT-qPCR. Results were normalized using GAPDH as gene control and expressed as means ± SEM (*n* = 6 per group per experiment, representative of two separate experiments) [^*∗*^*p* < 0.05 versus saline; ^#^*p* < 0.05 versus KO_2_ + vehicle (one-way ANOVA followed by Tukey's *t*-test)].

Detection of painful stimuli at the periphery by primary sensory neurons initiates and propagates the nociceptive signaling to spinal cord sites, where glial cells are activated to produce several mediators that induce nociceptor sensitization and neuroinflammation [30, 35]. Therefore, the mRNA expressions of glial cells activation markers were used to assess potential effect of tempol in inhibiting KO_2_-induced activation of spinal cord glial cells. Mice were treated with tempol (as for Figure 2) 40 min before KO_2_ intraplantar injection, and after additional 3 h spinal cord samples were collected for the determination of GFAP and Iba-1 mRNA expressions. GFAP and Iba-1 are markers of astrocytic and microglial activity, respectively (Figure 7). KO_2_ induced an increase of GFAP (Figure 7(a)) and Iba-1 (Figure 7(b)) mRNA expressions in the spinal cord, which was inhibited by tempol. These data indicate that tempol inhibits KO_2_-induced activation of glial cells in the spinal cord.

### 3.8. Tempol Inhibits Carrageenan- and CFA-Induced Hyperalgesia

**Figure 8:**
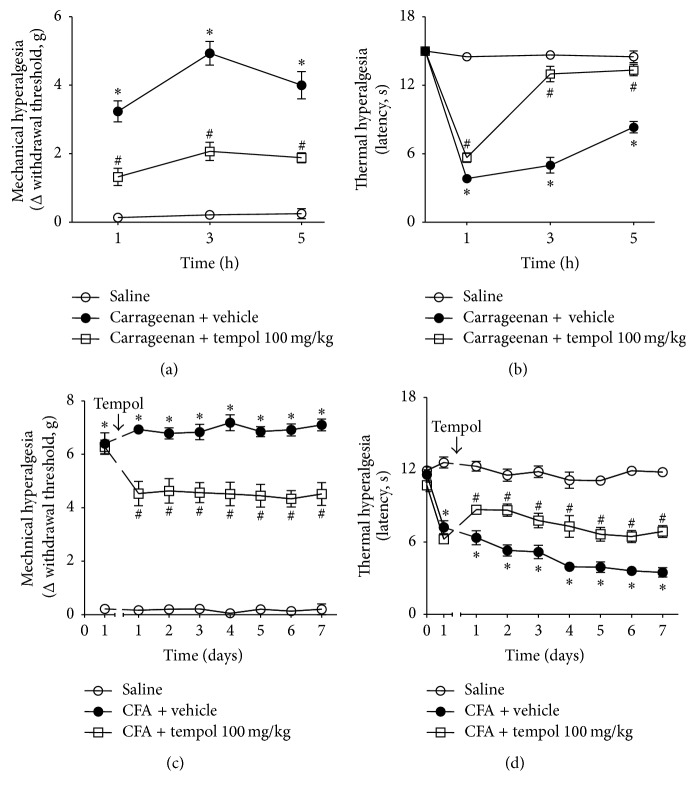
Tempol inhibits carrageenan and complete Freund's adjuvant- (CFA-) induced hyperalgesia. Mice were treated with tempol (100 mg/kg) or vehicle 40 min before carrageenan or CFA intraplantar injection. Nociceptive thresholds to mechanical and thermal stimuli were measured 1–5 h after carrageenan injection (panels (a) and (b), resp.) and 1–7 days after CFA injection (panels (c) and (d), resp.). Results are expressed as means ± SEM (*n* = 6 per group per experiment, representative of two separate experiments) [^*∗*^*p* < 0.05 versus saline; ^#^*p* < 0.05 versus carrageenan + vehicle for panels (a)-(b) and versus CFA + vehicle for panels (c)-(d) (ANOVA followed by Tukey's *t*-test)].

The ability of tempol to inhibit carrageenan- and CFA-induced mechanical hyperalgesia and thermal hyperalgesia was also tested considering that these are classical models of inflammatory hyperalgesia [23] (Figure 8). Mice were treated as described in the experimental protocol section. Tempol treatment inhibited carrageenan- (Figures 8(a) and 8(b)) and CFA- (Figures 8(c) and 8(d)) induced mechanical hyperalgesia and thermal hyperalgesia between 1 and 5 h and between 1 and 7 days, respectively. These results indicate that the analgesic effect of tempol is not limited to superoxide anion-triggered hyperalgesia, but it is also relevant in classical models of inflammatory pain such as carrageenan and CFA.

## 4. Discussion

Overproduction of superoxide anion has been implicated in excessive inflammatory response and pain by a mechanism in which superoxide anion nitrates manganese (Mn) SOD blocking its activity. As a consequence of this blockade, there are persistent high levels of superoxide anion with deleterious effects on tissues leading to increased nociceptive transmission [1–4]. In the present study, the effect of the SOD mimetic agent tempol over KO_2_ (superoxide anion donor)-induced pain and inflammation was evaluated. Tempol inhibited KO_2_-induced pain and inflammation. These effects were accompanied by inhibition of production of several proinflammatory and prooxidant mediators. Therefore, tempol emerges as an interesting molecule to efficiently target inflammatory pain scenarios related to superoxide anion. In agreement, the analgesic effect of tempol was also observed in the carrageenan and CFA classic models of inflammation.

There seems to be a strong interaction between inflammatory signaling and the amplification of superoxide anion production and vice versa. For instance, Toll-like receptor (TLR)/interleukin-1 receptor-associated kinase (IRAK) signaling leads to a fast activation of NADPH oxidase resulting in superoxide anion production [36, 37]. Superoxide anion mediates TLR4-induced nuclear factor *κ*B (NF-*κ*B) activation. NF-*κ*B is considered a redox-sensitive transcription factor [38]. In fact, NF-*κ*B activation has been implicated in de novo superoxide anion production [38] and TLR4/myeloid differentiation primary response gene 88 (MyD88) activation induces inflammatory pain [39]. Cytokines such as TNF*α* and IL-1*β* can activate NADPH oxidase inducing superoxide anion production [40, 41], and TNF*α*/TNFR1 signaling mediates superoxide anion-triggered pain and oxidative stress and TNF*α*-induced hyperalgesia depends on superoxide anion [7]. In addition to proinflammatory cytokines, there is the participation of anti-inflammatory cytokines. IL-10 is an antihyperalgesic cytokine in inflammatory, neuropathic, and cancer pain models [42–44]. For instance, IL-10 deficiency increases superoxide anion production and NADPH oxidase activity demonstrating that IL-10 limits inflammation and hyperalgesia [31, 45].

Nociceptors express cytokine receptors enabling cytokines to activate these neurons [30]. During peripheral inflammation, resident cells such as macrophages and mast cells and recruited cells such as neutrophils converge for producing large amounts of mediators including TNF*α*, IL-1*β*, and PGE_2_. These hyperalgesic molecules interact with their respective receptors in peripheral nerve terminals of primary nociceptive neurons triggering intracellular signaling pathways that lead to the opening of ion channels such as Na_v_1.7, Na_v_1.8, Na_v_1.9, transient receptor potential V1 (TRPV1), and TRPA1. The consequent depolarization and increased action potential generation and propagation in the peripheral sensory neuron toward central terminals result in hyperalgesia [30]. There is evidence that cytokines induce COX-2-dependent production of PGE_2_, which in turn sensitizes nociceptor neurons [29, 33]. During prostaglandin production, COX-2 also produces superoxide anion, and superoxide anion induces COX-2 expression and activity-dependent hyperalgesia [21, 38]. Therefore, the inhibition of KO_2_-induced TNF*α*, IL-1*β*, and COX-2 expressions by tempol is an important mechanism for inhibition of hyperalgesia. ET-1 is a hyperalgesic peptide [33], which mediates superoxide anion-induced pain, cytokine production, and oxidative stress [21, 22]. The present data show that tempol inhibited peripheral and spinal cord superoxide anion-induced TNF*α*, IL-1*β*, COX-2, and preproET-1 mRNA expressions, evidencing that tempol treatment reduces the production of a cascade of inflammatory molecules triggered by superoxide anion and that this mechanism might contribute to reducing superoxide anion-induced pain and inflammation.

It is also important to mention that superoxide anion activates terminal endings of nociceptive neurons through the redox-sensitive TRPA1 [10, 46] and reduces the threshold of nociceptive neurons through protein kinase (PK) C*ε*-mediated TRPV1 activation [47]. Superoxide anion can also directly activate nociceptive neurons to induce pain. Interestingly, neurotransmission seems to have a role in inducing superoxide anion production considering that glutamate triggers it [48]. The above data suggest that superoxide anion can induce direct and indirect effects in nociceptive transmission resulting in hyperalgesia via molecules such as cytokines, ET-1, and prostanoids [29].

Besides nitration of MnSOD, it is possible that superoxide anion induces its own production via NADPH oxidase. In fact, tempol reduced KO_2_-induced depletion of total antioxidant capacity and reduced the mRNA expression of the NADPH oxidase subunit gp91^phox^ and its product superoxide anion. Importantly, the superoxide anion production assessed by NBT method was observed 3 h after KO_2_ injection. In saline solution, superoxide anion vanishes in up to 10 min [20]. Therefore, it is unlikely that the superoxide anion production at peripheral and spinal cord levels observed 3 h after KO_2_ injection reflected the superoxide anion that was peripherally injected but rather an endogenous superoxide anion that was produced in response to the inflammatory stimulus. In this sense, it is reasonable to consider that, in addition to the accumulation of superoxide anion levels by the inhibition of MnSOD [49], immune cells produce superoxide anion contributing to increasing its levels.

Tempol acts catalyzing superoxide anion, which would increase hydroxyl radical (^•^OH) levels production from hydrogen peroxide (H_2_O_2_). However, tempol protects cells from oxidative damage given that it also presents catalase-like activity, generating H_2_O and O_2_ from H_2_O_2_ and inhibiting ^•^OH production [12, 17]. These effects explain reduced lipid peroxidation achieved after tempol treatment in several experimental models [15, 17–19]. Therefore, tempol is capable of modulating the activity of distinct components involved in the oxidative* milieu*, conferring additional protection to the tissues.

Nrf2 is a determinant transcription factor for the control of oxidative stress [34, 50]. KO_2_ reduced Nrf2 mRNA expression in the paw skin and spinal cord, whereas tempol recovered its expression at both sites. Evidence indicates that Nrf2 can induce IL-10 and GSH production [50]. Herein, tempol did not increase IL-10 mRNA expression but rather reduced the mRNA expression of TNF*α* and IL-1*β*, indicating that the mechanism of action of tempol does not include increasing IL-10 mRNA. Given that IL-10 is produced concomitantly to inflammatory cytokines to limit inflammation and pain [31], diminishing IL-10 mRNA expression can be a consequence of the tempol inhibition of superoxide anion-induced TNF*α* and IL-1*β* mRNA expressions. Tempol inhibited KO_2_-induced depletion of GSH levels, which corroborates the tempol-induced increase of Nrf2 mRNA expression.

Inflammatory response at peripheral sites increases the nociceptive inputs at the spinal cord. Spinal glial cells respond to increased neurotransmission producing inflammatory cytokines and free radicals, including superoxide anion [29, 30]. Considering that tempol is cell-permeable and has the ability to cross the blood-spinal cord barrier due to its low molecular weight, it is possible that the antihyperalgesic effects of tempol reported here also reflect its activity at the spinal cord level, what needs to be further investigated. Nevertheless, the peripheral inhibition of nociceptive input by tempol also leads to a reduction of neurotransmission and spinal cord glial cells activation [51]. It is reasonable that the overall reduction of KO_2_-induced inflammation by tempol at peripheral sites resulted in reduced sensitization of spinal cord neurons. The spinal cord glial cell markers GFAP and Iba-1 reflect the activation of astrocytes and microglia, respectively. These cells contribute to hyperalgesia by producing nociceptive molecules such as cytokines which amplify nociceptive transmission [35]. KO_2_ increased GFAP and Iba-1 mRNA expressions and peripheral treatment with tempol reduced their levels, suggesting that tempol inhibited spinal cord glial cells activation and consequently hyperalgesia.

Tempol inhibited carrageenan- and CFA-induced mechanical hyperalgesia and thermal hyperalgesia. These results support the applicability of tempol as an analgesic in other models of inflammation. Tempol is also an effective treatment in other pain conditions. For instance, tempol inhibits mechanical hyperalgesia and thermal hyperalgesia in models of neuropathic pain induced by CCI and chemotherapy [14–16]. Therefore, these data support the potential of tempol as a promising analgesic and anti-inflammatory molecule for the treatment of both inflammatory and neuropathic pain.

In the present study, we demonstrate that peripheral injection of a superoxide anion donor induces the upregulation of the NADPH oxidase subunit gp91^phox^ mRNA expression in the paw skin and spinal cord, which was reduced by the SOD mimetic agent tempol. Moreover, tempol reduced superoxide anion-induced TNF*α*, IL-1*β*, preproET-1, and COX-2 mRNA expressions in both paw skin and spinal cord. In fact, targeting these mediators in the paw skin and spinal cord with tempol or with other analgesics (present data, [2, 7, 20–22]) reduced superoxide anion-induced pain and inflammation. Finally, superoxide anion-triggered peripheral inflammation results in spinal cord activation of astrocytes and microglia, which was inhibited by tempol. This is important considering that long-term activation of these cells drives to a chronic pain state. Additionally, tempol inhibited not only superoxide anion-triggered inflammatory pain but also carrageenan- and CFA-induced inflammatory pain. Therefore, the present data suggest the importance of further investigation of tempol or tempol-like compounds in additional translational models of pain and/or chronic inflammation.
